# Development of a telehealth obesity OSCE and reliable checklist for assessment of resident physicians: a pilot study

**DOI:** 10.1186/s12909-022-03672-5

**Published:** 2022-08-19

**Authors:** Natalie A. Cameron, Robert F. Kushner

**Affiliations:** 1 Department of Medicine, Division of General Internal Medicine, Northwestern University, Feinberg School of Medicine, Chicago, USA; 2 Departments of Medicine and Medical Education, Division of Endocrinology, Metabolism and Molecular Medicine, Northwestern University, Feinberg School of Medicine, 645 North Michigan Avenue, Suite 530, Chicago, USA

**Keywords:** Objective structured clinical examination, Obesity, Telehealth, Inter-rater reliability, Medical education

## Abstract

**Background:**

Obesity is a major public health problem, yet residents undergo little formal training and assessment in obesity-related care. Given the recent growth of telehealth, physicians must further learn to apply these skills using a virtual platform. Therefore, we aimed to develop an objective structured clinical examination (OSCE) with reliable checklists to assess resident ability to take a patient-centered obesity-focused history that was feasible over telehealth based on published obesity competencies for medical education.

**Methods:**

We developed a 15-minute telehealth OSCE to simulate an obesity-related encounter for residents modified from a script used to assess medical student obesity competencies. We designed three checklists to assess resident skills in history taking, communication and professionalism during the obesity-related encounter. Resident performance was assessed as the percentage of obesity-related history taking questions asked during the encounter and as the mean communication and professionalism scores on a scale of 1 through 5 with 1 representing unacceptable/offensive behavior and 5 representing excellent skills. Encounters and assessments were completed by two commissioned actors (standardized patients) and 26 internal medicine residents over a secure online platform. We assessed the reliability of each checklist by calculating the percent agreement between standardized patients and the kappa (κ) statistic on each checklist overall and by each checklist item.

**Results:**

Overall agreement between standardized patients on the history taking, communication and professionalism checklists were 83.2% (κ = 0.63), 99.5% (κ = 0.72) and 97.8% (κ =0.44), respectively. On average, residents asked 64.8% of questions on the history taking checklist and scored 3.8 and 3.9 out of 5 on the communication and professionalism checklists, respectively.

**Conclusions:**

Results from this pilot study suggest that our telehealth obesity OSCE and checklists are moderately reliable for assessing key obesity competencies among residents on a virtual platform. Integrating obesity OSCEs and other educational interventions into residency curricula are needed to improve resident ability to take an obesity-focused history.

**Supplementary Information:**

The online version contains supplementary material available at 10.1186/s12909-022-03672-5.

## Background

Obesity, defined as a body mass index of 30 kg per meter squared or greater, is a major public health problem affecting more than 40% of adults in the United States [1]. This excess weight increases the risk of diabetes, coronary heart disease, stroke, high blood pressure, anxiety, depression and all-cause mortality among other conditions [2]. Several national organizations including the American Heart Association, The Obesity Society and United States Preventive Services Task Force recommend that physicians screen for obesity and help patients initiate and maintain weight loss via counseling and engagement in multicomponent behavioral interventions [3, 4]. Despite these recommendations, primary care providers incorporate weight management counseling into only 20% of patient appointments [5]. Although barriers to adequate obesity care exist at all levels of the medical system including lack of time and reimbursement, inadequate physician training remains an important obstacle [6, 7]. Currently, up to one-fifth of internal medicine training programs, which are responsible for training primary care physicians who are at the frontlines of obesity care, provide very little instruction on physical activity and nutrition, and more than one-third provide very little or no instruction on psychosocial and behavioral components of obesity, weight stigma and discrimination [8]. In a national survey of primary care physicians in the United States, approximately 90% identified additional training in nutrition and physical activity counseling as a targetable intervention to improve obesity related care [9]. In fact, physicians who learn “good obesity practices” in medical school and residency are more likely to recommend weight loss, discuss diet and exercise and refer patients to specialized weight related services [6]. Furthermore, obesity-related educational interventions have been shown to help patients lose weight and reduce obesity bias among practitioners [10, 11].

Given the rising obesity prevalence in the United States, reforms to medical education, particularly during residency, are needed to incorporate more comprehensive obesity-related training to improve the quality and quantity of obesity counseling and care in the primary care setting. The Objective Structured Clinical Examination (OSCE) is widely used as an important tool for teaching and assessing history taking, physical examination and communication skills [12]. During an OSCE, medical trainees engage in a simulated patient encounter with a trained actor (standardized patient [SP]) who portrays a patient with a specific concern and constellation of symptoms. Trainees are assessed by SPs or physician educators based on their ability to gather pertinent information regarding the medical concern and/or perform an appropriate physical exam. OSCEs and role-playing educational interventions have shown promise in improving obesity-related care [10]. Specifically, participation in a multi-modal obesity counseling curricula involving case-studies, role-playing and practice with SPs improved the quality of obesity counseling among primary care residents [13]. However, few studies have utilized obesity OSCEs [14–16], and there are no current standardized assessment tools for measuring obesity-related clinical skills among resident physicians. Furthermore, given the transformation of healthcare delivery and rise in telehealth during the COVID-19 pandemic, it is essential that training and assessment tools be developed on virtual platforms [17].

Therefore, we aimed to develop an obesity OSCE with an associated reliable checklist to assess internal medicine resident ability to take a patient-centered obesity-focused history that was feasible over telehealth based on the Obesity Medicine Education Collaborative’s obesity competencies for medical education [18].

## Methods

### Participants

#### Resident physicians

Post-graduate year (PGY) 2–3 internal medicine residents at an academic medical center were eligible to participate. PGY-1 resident physicians were excluded from participation given limited experience with ambulatory encounters and lack of schedule flexibility to participate in the study. A member of the study team recruited residents on elective rotations via email after approval from the residency program director and Vice Dean of Education. Of the 26 residents who provided written, online consent to participate, 23 consented to record their sessions for review by study investigators and SPs after the encounter. This educational study was exempt from Institutional Review Board approval.

#### Standardized patients

We recruited two SPs through the university’s Clinical Education Center to portray adult patients with obesity. SPs received hourly monetary compensation for participation with funds awarded to the study’s principal investigator from the Healthy Patient Initiative. Both SPs consented to OSCE recording.

### OSCE content and format

We adapted the OSCE script from an obesity OSCE used for medical student assessment at the university. The script detailed the chief complaint (weight gain), opening statement (“I am beside myself - I have gained so much weight - I want to get control over it”), and answers to resident questions regarding the patient’s obesity history, dietary patterns, physical activity, concerns about obesity and past medical history (Supplemental Fig. 1). We modified the script to include SP statements that challenged resident physicians (ex. “I am so frustrated I cannot keep off the weight….Is there something wrong with me?”) and adapted to the COVID-19 pandemic (ex. “I work from home”). Script modifications were discussed with faculty at the Clinical Education Center with experience conducting medical student and resident OSCEs.

Prior to study initiation, SPs attended a 1-hour virtual training session on the video platform with the investigators to review the script. SPs were also instructed on how to use several features of the video platform including changing their name to the patient’s name, adjusting the screen to display the resident, and ensuring sound and video worked appropriately.

OSCEs took place on the virtual platform and were moderated by a member of the study team. At the beginning of the encounter time, features of the video platform described in a guide emailed to the residents prior to the encounter were reviewed. The moderator then displayed a resident instruction sheet which included information on the goal of the encounter (“elicit an obesity focused history”), chief complaint and medical history. Residents were not expected to perform a physical exam or provide counseling. The participating SP then “entered the room” by turning on their sound and video to start the 15-minute OSCE. The second SP and moderator observed the encounter with sound and video off. At the end of the encounter, the participating SP provided the resident with approximately 5-minutes of verbal feedback.

### Resident assessment

The investigators created SP checklists, assessing three targeted competencies: resident history taking skills, communication skills and professionalism. Checklist items were based on the Obesity Medicine Education Collaborative’s obesity competencies for medical education [17]. The history taking checklist consisted of 14 Yes/No items assessing if residents asked obesity related history questions during the OSCE (Supplemental Fig. 2). Communication and professionalism skills were assessed using a 9-item and 6-item checklist, respectively, on a 5-point Likert Scale in which scores of 1 represented unacceptable or offensive behavior, 2 subpar but not offensive behavior, 3 acceptable skills, 4 above average skills and 5 excellent skills (Supplemental Figs. 3 and 4). Checklists were reviewed with several experts in medical education and assessment including a national leader and professor of medical education at the university, and an OSCE team with extensive experience conducting OSCEs at the university’s medical school. Checklist structure (i.e. Likert scale vs yes/no items) and content were revised based on their feedback during structured group meetings. Both the participating and observing SPs completed the checklists for each resident using a secure, virtual survey platform after the OSCE; residents completed modified versions of the checklists for self-assessment (Supplemental Figs. 5, 6 and 7).

As is typical in medical assessment, SPs were instructed on appropriate checklist completion during a pre-OSCE training session. To understand and resolve discrepancies in SP checklist assessments, we used an iterative process by meeting with SPs after completion of 10 resident encounters (mid-OSCE feedback) and at the conclusion of the study (post-OSCE debrief). Several clarifying statements and examples were added to the checklists after the mid-OSCE feedback session (Supplemental Figs. 2, 3 and 4). After all sessions were completed, SPs once again watched the recorded resident encounters (*n* = 23) and individually re-evaluated the residents using the checklists.

### Resident surveys

Resident participants completed a pre-OSCE survey for demographic information and a post-OSCE survey to assess the feasibility of the OSCE over telehealth using the following questions: 1) “Was this an acceptable format for you to conduct an OSCE?”; 2) “How realistic was it for you to evaluate a patient via this telehealth encounter?”; 3) “Please describe any technical challenges you faced during the encounter.”

### Statistical analysis

#### Inter-rater reliability

To assess inter-rater reliability among standardized patients, the percent agreement and kappa statistic (κ) were calculated for each checklist overall and on each checklist item after the post-OSCE debrief. To better understand the factors that contributed to discrepancies between SP ratings on several history taking checklist items, we conducted a post-hoc analysis comparing agreement between SPs on history taking items with less than 75% agreement by the SP performing the encounter. For the communication and professionalism checklist items, agreement was reached if SP assessments were within one Likert scale rating of each other.

#### Resident performance

Performance on the history taking checklist was assessed as the percent of residents who received credit for each checklist item averaged across SPs after the post-OSCE debrief. For communication and professionalism checklists, mean resident scores were calculated for each checklist item. Mean resident self-assessment scores were also calculated for each checklist item and compared with SP assessment scores using Student’s t-tests.

As a secondary analysis, we calculated mean resident performance on each checklist by telehealth experience (< 5 days vs > 5 days).

#### Qualitative assessment

We conducted a qualitative analysis of resident responses to the post-OSCE survey by calculating the percentage of residents who described the telehealth encounter as an acceptable format for assessment, who indicated that the encounter was realistic, and who experienced technical challenges. We also used SP feedback during the mid-OSCE feedback and post-OSCE debrief to propose modifications to our script and checklist for future use.

This study was funded by the Healthy Patient Initiative. This funding body played no role in the design of the study, data collection, data analysis, interpretation of the data or writing of the manuscript.

## Results

### Participants

Of the 26 resident participants, 42.3% were male, 69.2% PGY-2, 52.0% non-Hispanic white, 32.0% Asian/Pacific Islander and 4.0% non-Hispanic Black (Table 1). Most (64.0%) residents conducted <5 telehealth encounters prior to the OSCE.

**Table 1:** Descriptive statistics for resident participants

**N**	26
**Self-identified gender, N (%)**	
Male	11 (42.3)
Self-identified race/ethnicity, N (%)*	
Non-Hispanic White	13 (52.0)
Non-Hispanic Black	1 (4.0)
Hispanic/Latinx	0 (0.0)
Asian /Pacific Islander	8 (32.0)
Other	3 (12.0)
**Post-graduate year (PGY), N (%)**	
PGY2	18 (69.2)
PGY3	8 (30.8)
Days of telehealth experience, N (%)*	
None	3 (12.0)
1 through 5	13 (52.0)
6 though 10	6 (24.0)
11 or more	3 (12.0)

^*^N = 25 as one resident did not respond

Approximately half of participants were male, non-Hispanic White and had completed 1–5 telehealth encounters prior to OSCE participation. The majority of participants (69%) were post-graduate year 2 resident physicians

### Inter-rater reliability

Overall agreement on the history taking checklist was 83.2% (standard error [SE] = 2.1%) (κ = 0.63 [SE = 0.06]) (Table 2). Agreement ranged from 60.9% (SE = 10.4%) (κ = 0.23 [SE = 0.20]) on Item 8 (“Asked about barriers to healthy eating”) to 100.0% (SE = 0.0) (κ = 1.0 [SE = 0.21]) on Item 7 (“Asked about prior attempts to lose weight”). Out of the 14 checklist items, SPs achieved greater than 75% agreement on 9 items. The kappa statistic was greater than 0.4 for 7 out of 14 items indicating moderate to perfect agreement. For Items 1 and 9, percent agreement was high (87.0% [SE = 7.2%] and 95.7% [SE = 4.3%], respectively), while κ = 0.0 (SE = 0.0) for both items.

**Table 2:** Percent agreement (standard error) between standardized patients on each checklist overall and by checklist item

	N (Percent Agreement) [Standard Error]	Kappa Statistic (Standard Error)
**History Taking Checklist Overall**	***N***^†^ **= 321**	
	**267 (83.2) [2.1]**	**0.63 (0.06)**
**History Taking Checklist Items**	***N***^‡^ **= 23**	
1. Asked when the patient first began struggling with weight	20 (87.0) [7.2]	0.0 (0.0)
2. Asked the patient’s highest and lowest weights	22 (95.7) [4.3]	0.83 (0.21)
3. Asked about the patient’s past attempts to lose weight	22 (95.7) [4.3]	0.78 (0.20)
4. Asked why the patient thinks he/she is gaining/gained weight	17 (73.9) [9.4]	0.49 (0.20)
5. Asked a 24-hour diet recall	20 (87.0) [7.2)]	0.51 (0.18)
6. Asked about beverage consumption	21 (91.3) [6.0}	0.83 (0.21)
7. Asked about prior attempts to change his/her diet	23 (100.0) [0.0]	1.0 (0.21)
8. Asked about barriers to healthier eating	14 (60.9) [10.4]	0.23 (0.20)
9. Asked about the type of physical activity the patient performs	22 (95.7) [4.3]	0.0 (0.0)
10. Asked about the amount of physical activity the patient performs*	15 (68.2) [10.2]	0.31 (0.20)
11. Asked about barriers to performing more physical activity	15 (65.2) [10.2]	0.32 (0.20)
12. Asked about the patient’s concerns regarding his/her excess weight	18 (78.3) [8.8]	0.23 (0.13)
13. Asked if the patient feels supported by his/her family or partner	21 (91.3) [6.0]	0.83 (0.21)
14. Completed the encounter in the allotted time	17 (73.9) [9.4]	0.39 (0.20)
**Communication Skills Checklist Overall**	***N***^†^ **= 207**	
	**206 (99.5) [0.5]**	**0.72 (0.47)**
**Communication Skills Checklist Items**	***N***^‡^ **= 23**	
1. Eye contact	23 (100.0) [0.0]	1.0 (1.5)
2. Facial expressions	23 (100.0) [0.0]	1.0 (0.72)
3. Body language	23 (100.0) [0.0]	1.0 (1.0)
4. Language and vocabulary	23 (100.0) [0.0]	1.0 (4.6)
5. Attention	23 (100.0) [0.0]	1.0 (3.2)
6. Verbalizing understanding of history	23 (100.0) [0.0]	1.0 (0.0)
7. Asking open ended questions	22 (95.7) [4.3]	−0.10 (0.81)
8. Using people first language	23 (100.0) [0.0]	1.0 (0.0)
9. Organization	23 (100.0) [0.0]	1.0 (1.9)
**Professionalism Checklist Overall**	***N***^†^ **= 138**	
	**135 (97.8) [1.2]**	**0.44 (0.37)**
**Professionalism Checklist Items**	***N***^‡^ **= 23**	
1. Respect	22 (95.7) [4.3]	−0.10 (0.89)
2. Empathy	22 (95.7) [4.3]	0.28 (0.69)
3. Honesty/integrity	23 (100.0) [0.0]	1.0 (1.47)
4. Responsibility/accountability	23 (100.0) [0.0]	1.0 (0.91)
5. Promoting a collaborative environment	22 (95.7) [4.3]	0.43 (0.58)
6. Demonstrating lack of bias	23 (100.0) [0.0]	1.0 (1.74)

†Total number of checklist items answered by standardized patients for all residents for entire checklist

‡Number of residents evaluated on each checklist item

* *N* = 22 as one SP did not respond to the item

Overall agreement on the history taking, communication and professionalism checklists were 83.2% (κ = 0.63), 99.5% (κ = 0.72) and 97.8% (κ = 0.44), respectively

Post-hoc analysis revealed that for 4 out of the 5 history taking items with less than 75% agreement, inter-rater agreement tended to be higher when SP1 participated in the encounter. Agreement on these four items ranged from 70.0% (SE = 15.3%) (κ = 0.40 [SE = 0.31]) to 90.0% (SE = 10.0%) (κ = 0.78 [SE = 0.31]) for SP1 and from 53.9% (SE = 14.4%) (κ = 0.11 [SE = 0.22]) to 61.5% (SE = 14.0%) (κ = 0.27 [SE = 0.20]) for SP2. However, these differences were not statistically significant (p > 0.05).

Overall agreement on the communication and professionalism checklists were 99.5% (SE = 0.5%) (κ = 0.72 [SE = 0.47]) and 97.8% (SE = 1.2%) (κ = 0.44) [SE = 0.37]), respectively, and ranged from 95.7–100% for each checklist item (Table 2). The kappa statistic was less than 0 on communication Item 7 (“Asked open ended questions”) and professionalism Item 1 (“Respect”) despite 95.7% agreement on both items.

### Resident performance

On average, residents asked 64.8% (SE = 1.2%) of items on the history taking checklist as assessed by SPs after the post-OSCE debrief (Table 3). Performance varied by checklist item ranging from 15.2% (SE = 5.4%) for asking the patient’s highest and lowest weights to 97.8% (SE = 2.2%) for asking about the type of physical activity the patient performs. Less than 50% of residents received credit on 6 out of 14 history taking checklist items. Average resident performance was 3.8 (SE = 0.0) and 3.9 (SE = 0.0) out of 5 on the communication and professionalism checklists, respectively (Table 3).

**Table 3:** Average resident performance on each checklist overall and each checklist item

	Standardized Patient Assessment	Resident Self-Assessment
**History Taking Checklist, N (%) [SE]**	***N*** **= 643**	***N*** **= 359**
	417 (64.8) [1.2]	258 (71.9) [2.4]*
**History Taking Skills Checklist Items**	***N*** **= 46**	***N*** **= 26**
1. Asked when the patient first began struggling with weight	43 (93.5) [3.7]	23 (92.0) [5.5]
2. Asked the patient’s highest and lowest weights	7 (15.2) [5.4]	2 (7.7) [5.3]
3. Asked about the patient’s past attempts to lose weight	41 (89.1) [4.6]	23 (92.0) [5.5]
4. Asked why the patient thinks he/she is gaining/gained weight	22 (47.8) [7.4]	12 (46.2) [10]
5. Asked a 24-hour diet recall	39 (84.8) [5.4]	21 (80.8) [7.9]
6. Asked about beverage consumption	22 (47.8) [7.4]	14 (53.8) [10)]
7. Asked about prior attempts to change his/her diet	40 (87.0) [5.0]	21 (80.8) [7.9]
8. Asked about barriers to healthier eating	27 (58.7) [7.4]	19 (73.1) [8.9]*
9. Asked about the type of physical activity the patient performs	45 (97.8) [2.2]	26 (100.0) [0.0]
10. Asked about the amount of physical activity the patient performs	16 (35.6) [8.3] ^†^	25 (96.2) [3.8]*
11. Asked about barriers to performing more physical activity	22 (47.8) [7.2]	17 (65.4) [9.5]*
12. Asked about the patient’s concerns regarding his/her excess weight	39 (84.8) [5.4]	19 (82.6) [8.1] ^‡^
13. Asked if the patient feels supported by his/her family or partner	22 (47.8) [7.4]	10 (38.5) [9.7]
14. Completed the encounter in the allotted time	32 (69.6) [6.9]	26 (100.0)[0.0]*
**Communication Skills Checklist Overall, Mean (SE)**	**3.8 (0.0)**	3.9 (0.1)*
**Communication Skills Checklist Items, Mean (SE)**		
1. Eye contact	4 (0.1)	n/a^§^
2. Facial expressions	3.7 (0.1)	n/a^§^
3. Body language	3.9 (0.1)	n/a^§^
4. Language and vocabulary	3.8 (0.1)	4.2 (0.1)*
5. Attention	3.9 (0.1)	3.8 (0.1)
6. Verbalizing understanding of history	3.7 (0.1)	4.1 (0.1)*
7. Asking open ended questions	3.9 (0.1)	3.9 (0.2)
8. Using people first language	3.9 (0.0)	4 (0.2)
9. Organization	3.6 (0.1)	3.6 (0.2)
**Professionalism Checklist Overall, Mean (SE)**	**3.9 (0.0)**	4.1 (0.1)*
**Professionalism Checklist Items, Mean (SE)**		
1. Respect	4.0 (0.1)	4.2 (0.2)
2. Empathy	4.0 (0.1)	4.2 (0.1)
3. Honesty/integrity	3.9 (0.1)	4.3 (0.1)*
4. Responsibility/accountability	3.8 (0.1)	3.8 (0.1)
5. Promoting a collaborative environment	3.8 (0.1)	4.1 (0.1)
6. Demonstrating lack of bias	3.9 (0.1)	n/a^§^

* *p* < 0.05 for t-test comparing standardized patient and resident self-assessment

^†^*N* = 45 given missing SP data point

^‡^*N* = 25 given missing resident data point

^§^ Item not included on resident self-assessment checklist

Residents asked 65–70% of items on the history taking checklist with <50% receiving credit on 6/14 items. Overall performance on communication and professionalism checklists ranged from 3.8–4.1/5

Resident self-assessment scores on the history taking checklist items were higher than SP assessment scores for the overall history taking checklist and for several communication and professionalism checklist items (Table 3). However, for Likert Scale ratings, resident and SP assessments did not differ by more than 0.5 points.

Residents who participated in >5 telehealth encounters prior to the OSCE had higher scores on the history taking checklist for both SP and resident self-assessments, and higher scores on the professionalism checklist for self-assessments (Table 4).

**Table 4:** Resident performance by telehealth experience

	**<** 5 telehealth encounters	> 5 telehealth encounters	***p***-value (t-test)
**Resident Self-Assessment**			
History Taking, % (SE)	67.6 (3.2)	79.4 (3.6)	0.02
Communication, Mean (SE)	3.8 (0.1)	4.1 (0.1)	0.10
Professionalism, Mean (SE)	3.9 (0.1)	4.5 (0.1)	0.00
**Standardized Patient Assessment**			
History Taking, % (SE)	61.7 (3.0)	72.6 (4.1)	0.04
Communication, Mean (SE)	3.8 (0.0)	3.8 (0.1)	0.78
Professionalism, Mean (SE)	3.9 (0.0))	3.9 (0.1)	0.73

Compared with residents who conducted <5 telehealth encounters prior to participation in the OSCE, residents who conducted >5 asked more history taking items during the OSCE

### Qualitative assessment

Of the 26 resident participants, 24 (92.3%) indicated that telehealth was an “acceptable” platform for the obesity OSCE and that the OSCE was either “realistic” or “worked well.” Of those who thought it was realistic, 8 residents (33.3%) stated that the encounter was either “very” or “extremely realistic.” Two residents commented that it was challenging not to provide counseling during the encounter. Technical issues were noted by 9 residents (34.6%), 8 of which were related to video freezing, although sound remained intact.

During the mid-OSCE feedback session and post-OSCE debrief, SPs commented that they were confused regarding how specific resident questions needed to be asked in order to receive credit for a history taking item. For example, regarding history taking Item 1 (“Asked when the patient first began struggling with weight”) SPs were unsure if the resident should receive credit for inquiring about a general time frame for weight gain (ex. years vs weeks) or if the resident needed to ask a specific age when weight gain began. There was also confusion in rating history taking items when SPs gave away the answer to a resident question during the OSCE before a resident formally asked the question.

## Discussion

In this pilot study we designed and implemented an OSCE and checklist to assess medical resident ability to take a patient-centered obesity-focused history using telehealth. This is the first obesity OSCE to assess resident performance using obesity competencies for medical education published by the Obesity Medicine Education Collaborative via telehalth [18]. Given that overall agreement between SPs on our history taking, communication and professionalism checklists were 83.2% (κ = 0.63), 99.5% (κ = 0.72) and 97.8% (κ = 0.44), our checklists are moderately to substantially reliable [19] for assessing overall performance via telehealth. Our results also revealed that residents neglected to ask several questions during the OSCE that are essential to guiding obesity-related counseling and management decisions in the primary care setting. These gaps should be addressed with curricular changes in medical education.

Although previous work has validated OSCEs for medical student and resident clinical skills [20–23], our OSCE is unique in its focus on obesity-related clinical skills via telehealth. Obesity is a major public health problem, yet less than half of physicians address weight management during primary care visits partially due to lack of training in obesity-related care [5, 6]. Therefore developing validated training and assessment tools for obesity competencies is essential to improving obesity-related care. In the current study, we present an OSCE and checklist with moderate to substantial reliability for the assessment of obesity related competencies. Additionally, our OSCE was feasible over a video based platform. Incorporating telehealth into residency training is an important next step in medical education given the rise in telehealth during the COVI-19 pandemic.

Although agreement between SPs on our checklists overall was 80–100%, agreement remained <75% on several history taking checklist items following the post-OSCE debrief. Discussion with SPs during the mid-OSCE feedback session revealed that these discrepancies were most likely due to differences in interpretation of checklist items, as well as uncertainty regarding if residents should get credit for asking a history taking item if SPs provided residents with the answers without specifically being asked. In addition, in our post-hoc analysis we found that agreement tended to be higher during encounters with SP1 who, qualitatively, offered more focused answers to resident questions than did SP2. These findings highlight the importance of pre-OSCE training that targets SP script and assessment interpretation, as well as continuous education, SP feedback and check-ins throughout the duration of the sessions to ensure consistency and SP retention as recommended by the Association of Standardized Patient Educators [24, 25].

For several items on our checklists, percent agreement was high (> 85%) while the kappa statistic was low (< 0). We suspect that this paradox may be related to the rare occurrence of a null response on these history taking items given that that the kappa statistic is associated with the prevalence of the finding and may not always be reliable for rare events [19]. For the communication and professionalism checklists, these discrepancies may have resulted from a high expected agreement given the weighting of the kappa statistic to allow SP answers to vary by 1 point on the Likert scale.

Despite discrepancies in SP ratings, our history taking assessment revealed consistently poor performance on several checklist items. Residents asked 65% of items on the history taking checklist; less than 20% asked the patient’s highest and lowest weight, and less than 50% asked about beverage consumption, family support and the patient’s perspective on weight gain. These results are consistent with prior studies revealing poor performance on obesity, nutrition and physical activity knowledge assessments among residents [26]. Interestingly, resident self-performance ratings tended to be higher than SP ratings. A prior review of the validity and accuracy of health assessments in medical education suggests that clinical self-performance ratings may be related to opinions regarding prior knowledge and abilities. Therefore, self-assessment is likely a trained skill like any other form of assessment [27]. This highlights the need for independent raters and further development of OSCEs for clinical assessment.

Despite overall higher resident self-assessments, performance remained low overall on the history taking checklist. Given the rising prevalence of obesity in the United States [28], and the importance of taking an obesity-focused history [29], it is essential that physicians be better trained in these skills. Interventions in medical education have shown promise in improving medical student and resident performance in obesity-related care [15]. However, more widespread, structured curricular changes are needed to improve obesity counseling among primary care physicians. In addition, since residents with more telehealth experience in our study tended to perform better on our obesity checklists, incorporating telehealth into obesity curricula may further prepare physicians to provide obesity-related care via telehealth.

### Strengths and limitations

Strengths of this study include the use of a telehealth platform to conduct the OSCE. The rise in telehealth during the coronavirus pandemic has required that physicians become more facile in delivering medical care over a virtual platform [17]. Our OSCE offers an opportunity for residency programs to incorporate telehealth into medical training. In addition, conducting our OSCE over telehealth allowed for compliance with social distancing recommendations and, therefore, the continuation of medical resident education and assessment in the setting of a global pandemic. Furthermore, compared with conventional in-person OSCEs, telehealth assessments are less costly and time-consuming as they reduce travel time, staffing and equipment needs [30]. This was also the first OSCE to use the Obesity Medicine Education Collaborative’s obesity related competencies to assess resident skills in obesity care [18].

Limitations of this study include a small sample size of resident physicians from an internal medicine residency program at a single institution and use of only two SPs for resident assessment. However, we were able to recruit up to one-third of the entire PGY-2 and PGY-3 residency class during the coronavirus pandemic. In addition, there were discrepancies between our SPs in script interpretation and OSCE performance, which may have contributed to differences in SP checklist assessments. Additional SP training ensuring consistency in SP performance and resident assessment prior to OSCE implementation could improve the reliability of our checklists for future use. Future research is needed to validate our checklists at other residency programs including other specialties that engage in obesity-related care (i.e. family medicine and pediatrics) using different SPs.

## Conclusions

In this pilot study, we present a feasible and reliable OSCE and checklist to assess resident ability to take a patient-centered obesity-focused history using established obesity competencies over telehealth. If validated at other institutions, our OSCE and checklist could be used as a standard assessment tool for obesity related history taking skills in medical education. As demonstrated in our study, validation will require thorough SP training and continuous feedback. Furthermore, our OSCE revealed several gaps in resident obesity-related competencies that must be addressed with structured curricular changes in medical education.

## Supplementary Information

**Additional file 1: Supplemental Figure 1**. Obesity OSCE Script. **Supplemental Figure 2**. History taking checklist for standardized patient assessment of resident physicians. **Supplemental Figure 3**. Communication checklist for standardized patient assessment of resident physicians. **Supplemental Figure 4**. Professionalism checklist for standardized patient assessment of resident physicians. **Supplemental Figure 5**. History taking checklist for resident self-assessment. **Supplemental Figure 6**. Communication checklist for resident self-assessment. **Supplemental Figure 7**. Professionalism checklist for resident self-assessment.

## Data Availability

The datasets generated and analyzed during the current study are available from the corresponding author on reasonable request.

## Abbreviations

OSCE: Objective structured clinical examination
SP: Standardized patient
