# Supplementary material for: Development of a telehealth obesity OSCE and reliable checklist for assessment of resident physicians: a pilot study
Source: BMC Med Educ. 2022 Aug 19;22:630. doi: 10.1186/s12909-022-03672-5 (PMC9389479; doi:10.1186/s12909-022-03672-5)
Supplement: Supplementary file 1 — Additional file 1: Supplemental Figure 1. Obesity OSCE Script. Supplemental Figure 2. History taking checklist for standardized patient assessment of resident physicians. Supplemental Figure 3. Communication checklist for standardized patient assessment of resident physicians. Supplemental Figure 4. Professionalism checklist for standardized patient assessment of resident physicians. Supplemental Figure 5. History taking checklist for resident self-assessment. Supplemental Figure 6. Communication checklist for resident self-assessment. Supplemental Figure 7. Professionalism checklist for resident self-assessment. [file 12909_2022_3672_MOESM1_ESM.docx]

**Supplemental Figure 1. Obesity OSCE Script**

**Department of Medicine Northwestern University Feinberg School of Medicine**

**Internal Medicine Residency Observed Structured Clinical Examination**

**Diagnosis:** Obesity

**PRESENTATION SITUATION:**

The patient presents to discuss his/her concerns about weight. The patient’s health insurance changed after his/her spouse switched companies, so he/she can no longer see his/her former PCP. The patient is planning to eventually establish care with this physician, but wants to focus on his/her weight today.

**General Appearance**: Casually dressed in street clothes, but well-groomed.

**Mood/Affect/Emotions**: Frustrated with self and concerned; looking for help.

Please note, superscripted numbers correlate to the item number on the history taking checklist.

**INTRODUCTION**

**Opening Statement:**

- I am beside myself. I have gained so much weight. I want to get control over it.

**If asked to “tell me more about that:”**

- Well, I know my weight is a problem. My last doctor told me that. I think it’s finally time to lose some weight.

**If asked again to “tell me more:”**

- What do you want to know?

**If asked what your previous doctor told you about your weight:**

- She told me that excess weight can cause diabetes. My dad has diabetes so I’m worried about that.
- Proposed revision to replace above statement with the following statement to avoid providing information about concerns regarding weight gain: “I don’t think we really talked about it very much”

**If asked why your weight is of particular concern today (versus sometime in the past):**

- I have never been happy about my weight, but now it is interfering with my ability to play with my kids. I tried to go down the slide with my son and could not fit. It was so embarrassing.^1^

**HISTORY OF PRESENT ILLNESS:**

Patterns of weight gain

- I have been overweight for as long as I can remember. I was actually bullied quite a bit in high school.^1^
- I gained another 20 pounds when I was in college. Then, I continued to put on weight to what I am now, which I think is the heaviest I’ve been.
- My lowest weight was *** pounds and my highest weight was *** pounds (provide numbers that are true for you)^2^

Reasons for weight gain^4^

- I started eating out a lot and drinking several times per week in college. I have now cut down to only 3 drinks or so on the weekends. I still usually get take out for lunch and order in food for dinner most days which I know is not good for my weight.
- I also think I gained almost 20 pounds after starting Paroxetine in college. Can that happen? Was my other doctor wrong for putting me on that medication?

Previous attempts to lose weight^3^

- I lost about 10 pounds on two occasions, but then gained back all the weight after returning to my previous habits.
- I am so frustrated that I can’t keep off the weight. Why can’t I do it? Is there something wrong with me?

Concerns about excess weight^12^

- I have two big concerns. First, my weight is now interfering with my ability to play with my kids. I tried to go down the slide with my son and could not fit. It was so embarrassing. Also, I know the weight is not good for my health. My dad had diabetes and my previous doctor said the excess weight can cause diabetes. Is this true? Am I going to get diabetes?

Diet

- Opening statement: I’m not consistent at all, so it’s really hard to say what my diet is like.
- If specifically asked about breakfast/lunch/dinner^5^:
  - Breakfast: Usually I just quickly eat a granola bar or Pop Tart. Sometimes I skip breakfast.
  - Lunch: I work from home, but most of the time I take a quick break to pick up take out from the local food chains like Chipotle, Subway or Panera. Sometimes I buy a salad with either Caesar or Ranch dressing if I want to be healthy. At least I do that, right?
  - Dinner: Most days we order in from the local pizza place.
- Snacks/Drinks^6^
  - I may snack on popcorn before I go to sleep.
  - For drinks, it is usually water or diet soda.
- Prior attempts to change diet^7^
  - I’ve tried a low carb diet and Slim Fast drinks, which both worked, but then gained back all the weight after returning to my previous eating habits.
- Barriers to healthy eating^8^
- Time is the biggest barrier. Although I work from home, I work for a startup and the work seems endless. My boss emails me all the time with updates and new projects. Between work and my kids, it is hard to find time to cook or go to the grocery store. Plus, I’m a terrible cook – the best I can do is Mac and Cheese from the box.
- Patient’s perception about his/her diet
  - I guess it’s not the best. I know I should make more home cooked meals. I should probably eat more vegetables too.
  - Positive aspects of diet: I sometimes eat granola bars in the morning and Caesar salads for lunch.
  - Negative aspects of diet: I eat out a lot for lunch and order in most days for dinner. We eat a lot of cheese pizza for dinner.
- Access to healthy food
  - Both my husband and I have stable jobs, so money is not an issue. We can afford healthy food.

Physical activity

- My husband/wife and I sometimes take walks on the weekends.^9^
  - Proposed revision to replace above statement with “My husband/wife and I sometimes take walks”
- We walk a few times around the block. I’m not really sure how far that is or how fast we go. It takes us about 30 minutes. We only walk on the weekends.^10^
- Barriers to increasing physical activity^11^
  - I don’t have a lot of free time. I already feel bad about working at my computer all day instead of spending quality time with my husband and kid.

Social Support^13^:

- My husband/wife is my main source of support. He/she also struggles with his/her weight and wants to lose weight too. We’re in it together

**PAST MEDICAL HISTORY**

- Pre-diabetes:
  - I was told 2 years ago by my doctor that my blood sugar was a little elevated after my fasting blood test. My doctor said we would just watch it. Do you think I am going to get diabetes?
- Depression:
  - I’m on Paroxetine which I have been taking since college when I started feeling depressed. I saw a psychologist for a few years, but I stopped about 5 years ago when I started consistently feeling good. I just take my medication now.
    - Proposed revision to add the following instructions: “Do not mention Paroxetine causing weight gain unless specifically asked about reasons for weight gain.”
  - If asked if you have little interest or pleasure in doing things: No
  - If asked if you are feeling down, depressed or hopeless: No. I just get a little down when I think about all this weight I have to lose, but it doesn’t last.

**PAST SURGICAL HISTORY:** None

**MEDICATIONS:**

- Paroxetine 20mg daily
- If asked about reasons for why you think you have gained weight: I think I gained almost 20 pounds after starting Paroxetine in college. Can that happen? Was my other doctor wrong for putting me on that medication?
  - Proposed revision to add the following instructions: “Do not mention Paroxetine causing weight gain unless specifically asked about reasons for weight gain.”

**ALLERGIES:** None

**FAMILY HISTORY:**

- Father: Type 2 Diabetes, Obesity
- Mother: Hypertension, Obesity
- Siblings: None

**SOCIAL HISTORY**

- Married with 1 child who is three years old. He/she has not yet started pre-school and does not go to day care. Husband works in law enforcement and has a steady income.
- Works from home as a website developer
- Alcohol: Drank several times per week in college. Now drinks approximately 3 drinks on weekends.
- Tobacco: Never smoked
- Drugs: Never tried marijuana, cocaine or IV drugs

**REVIEW OF SYSTEMS:** Otherwise feeling

**Supplemental Figure 2. History taking checklist for standardized patient assessment of resident physicians**

Participant Study Number _____

SP participant study number ______

|  | **0** | **1** |
| --- | --- | --- |
| 1. **Asked when the patient first began struggling with weight**   *Ex. When did you start putting on weight?*  *When did you first become unhappy with your weight?*  *For how long have you been struggling with your weight?*  *If resident asks: “ What has your weight trend been?”, ask for clarification^*^*  *The resident should be able to elicit a response that describes a general time frame for*  *weight gain (slow or sudden, years vs months)^*^* | **No** | **Yes** |
| 1. **Asked the patient’s highest and lowest weights** | **No** | **Yes** |
| 1. **Asked about the patient’s past attempts to lose weight**   *Ex.* *What have you done in the past to try to lose weight?*  *Have you been successful at losing weight in the past?*  *Have you tried any weight loss programs?*  *Would give credit if ask about both past attempts to change diet AND exercise* | **No** | **Yes** |
| 1. **Asked why the patient thinks he/she is gaining/gained weight**   *Ex. Why do you think you gained weight?*  *What do you attribute your weight gain to?*  *What was going in your life when you gained the weight?*  *Were you taking any medications that may have caused weight gain?*  *If resident inquires about or mentions medications and SP offers information about*  *Paroxetine causing weight gain, then the resident should be given credit for this*  *question even if they do not ask about other potential reasons for weight gain^*^* | **No** | **Yes** |
| 1. **Asked a 24-hour diet recall**   *Ex. What do you typically eat in a day, or an example of a day?*  *What do you typically eat for breakfast, lunch and dinner?*  *Please let me know what you had to eat yesterday?* | **No** | **Yes** |
| 1. **Asked about beverage consumption**   *Ex. Do you drink any sugar-sweetened beverages?* | **No** | **Yes** |
| 1. **Asked about prior attempts to change his/her diet**   *Ex. Have you tried to change your diet in the past?*  *What have you tried to do to change your diet/eating habits in the past?*  *Have you ever been successful changing your diet?*  *Have you tried [Weight Watchers, meal delivery services etc] in the past?*  *Have you ever seen a registered dietitian or entered a commercial weight loss program?*  *The resident should get credit for this question and Question 3 if he/she asks about prior*  *attempts to lose weight (Question 3) and the SP responds regarding dietary changes^*^* | **No** | **Yes** |
| 1. **Asked about barriers to healthier eating**   *Ex. What are the biggest challenges you face in changing your diet?*  *What did you struggle with when you tried to change your diet in the past?*  *What is gets in the way of choosing a healthier diet?*  *Would give credit for specific questions such as:*  *Do you have time to prepare meals? Can you afford healthy foods?*  *What makes it hard to change your diet?^*^* | **No** | **Yes** |
| 1. **Asked about the type of physical activity the patient performs**   *Ex. How do you stay active?*  *What type of exercise do you do?*  *What is the most active thing you do?*  *If asks “Do you exercise?,” needs to follow up with a more specific question. Do not give any*  *additional information at this time (that is item 10)* | **No** | **Yes** |
| 1. **Asked about the amount of physical activity the patient performs**   *Ex. For how many minutes per day/hours per week do you stay active?*  *How often or for how long to you get your heart rate up?* | **No** | **Yes** |
| 1. **Asked about barriers to performing more physical activity**   *Ex. Is there anything that prevents you from being more active?*  *What is your biggest barrier to being more active?*  *Based on one of your prior responses, they could say:*  *“It sounds like [time] is your biggest barrier. Is that correct?”*  *“Is it difficult to exercise because of [barrier you mentioned in another question]”^*^* | **No** | **Yes** |
| 1. **Asked about the patient’s concerns regarding his/her excess weight**   *Ex. Is there something that particularly concerns or bothers you about your weight?*  *Do you have any worries about your weight?*  *Why do you want to lose weight?*  *What is it about your weight that brought you in today?^*^*  *What particularly brought you in today (vs another day). What was the tipping point?^*^*  *Note: Do not give credit if the resident only asks “What brought you in today?”^*^* | **No** | **Yes** |
| 1. **Asked if the patient feels supported by his/her family or partner**   *Asks if other family members are struggling with a similar problem and SP states that the family member is supportive^*^* | **No** | **Yes** |
| 1. **Completed the encounter in the allotted time and provided closure^†^**   *Resident is able to provide closure at the end of the encounter. Ex. “It was a*  *pleasure seeing you today. “Let’s follow up in [a certain amount of time]”^*^*  *~~Resident acknowledges that the encounter is complete prior to the time limit~~^‡^* | **No** | **Yes** |

* Item added after mid-OSCE feedback

^†^ Statement “and provided closure” was added after mid-OSCE feedback

**^‡^** Statement was eliminated at mid-OSCE feedback

**Supplemental Figure 3. Communication checklist for standardized patient assessment of resident physicians**

Participant Study Number _____

SP participant study number ______

Please rate the resident’s communication using the following rating scale^*^:

1 = Unacceptable or offensive communication

2 = Subpar, but not offensive communication

3 = Acceptable communication

4 = Above average communication

5 = Excellent communication. Cannot think of any suggestions for improvement.

|  | **1** | **2** | **3** | **4** | **5** |
| --- | --- | --- | --- | --- | --- |
| 1. **Eye Contact** | Uses minimal eye contact/is more focused on notes than on patient/uses uncomfortable amount of eye contact |  | Makes eye contact that fosters patient connection most of the time |  | Makes consistent eye contact which fosters patient connection throughout the interview |
| 1. **Facial Expressions** | Facial expressions (or lack thereof) creates a disconnect |  | Facial expressions convey interest/engagement at times (e.g. nods, changes expression appropriately in response to information) |  | Facial expressions convey consistent, genuine, appropriate interest and engagement throughout the encounter |
| 1. **Body Language** | Has distracting body language throughout the encounter |  | Body language fosters connection. Very few distractions |  | Body language greatly facilitates connection (e.g. angles towards patients, leans forward, no distractions) |
| 1. **Language and Vocabulary** | Uses medical jargon frequently without explanation or uses slang that is jarring |  | Usually explains medical terms when using them; can always explain terms if prompted |  | Avoids medical terminology or explains without prompting, adjusts information to suit patient’s level of understanding |
| 1. **Attention** | Seems to ignore or forget information provided by patient, often repeats questions |  | Asks/duplicates very few questions and usually explains or acknowledges repeated questions |  | Does not repeat questions without explanation or acknowledgement. Questions build off information the patient provides |
| 1. **Verbalizes understanding of history** | Does not verbalize their understanding of patient’s history |  | Reflects, clarifies, or summarizes the history at times during the interview |  | Reflects, clarifies, or summarizes the history frequently during the interview |
| 1. **Open Ended Questions** | Almost exclusively uses closed ended questions/does not use facilitating remarks^*^ |  | Uses facilitating remarks or open-ended questions at points in the interview |  | Uses many facilitating remarks and open-ended questions throughout the interview |
| 1. **People First Language** | Rarely uses people first language |  | Uses people first language at times during the interview |  | Almost always uses people first language during the interview |
| 1. **Organization** | The encounter was disorganized. Did not use transition phrases or sign-posting^‡^ |  | The encounter was mostly organized. Occasionally used transition phrases and sign-posting |  | The encounter flowed easily and logically from one topic to the other, facilitated by the consistent use of transition phrases and sign-posting |

**Description of Open Ended Questions**

- Open ended questions are those that cannot be answered with a “yes” or “no” response and encourage elaborative responses from the interviewee
- Examples of “facilitative remarks” include: “Tell me more about that;” “What else has been happening?”

**Description of “people first language”**

- “People first language” refers to a sentence structure that avoids labeling and defining individuals by their medical condition by naming patients first and their diagnoses second. Using this sentence structure helps separate patients from their diagnoses and allows providers to focus on their patient as a whole, rather than defining them by their diagnoses.

| Examples of people first language | Examples of non-people first language |
| --- | --- |
| - “patients living with obesity,” “patients with excess weight” - “How long have you struggled with your weight?” - “Why do you think you struggle with your weight?” - “Are there any other family members with obesity?” | - “obese people,” “fat people” - “How long have you been/when did you first become obese?” - “Why do you think you are obese/fat?” - “Are there any other obese family members?” |

**Description of sign posting**

- Language used by the provider to inform the patient what will be said or done next. For example “Now that we have talked about xxx, we will discuss xxx next…”

^*^Item added after mid-OSCE feedback

**Supplemental Figure 4. Professionalism checklist for standardized patient assessment of resident physicians**

Participant Study Number _____

SP participant study number ______

Please rate the resident’s professionalism using the following rating scale:^*^

1 = Unacceptable or offensive

2 = Subpar, but not offensive

3 = Acceptable

4 = Above average

5 = Excellent. Cannot think of any suggestions for improvement.

|  | **1** | **2** | **3** | **4** | **5** |
| --- | --- | --- | --- | --- | --- |
| 1. **Respect** | Lacks professional demeanor during history (e.g. minimally acknowledges patient beyond chief concern) |  | Usually acknowledges patient beyond chief concern |  | Consistently interacts with patient using utmost respect (e.g. shows a lot of interest in patient beyond chief concern) |
| 1. **Empathy** | Does not offer verbal or nonverbal empathetic responses to patient’s stress or distress |  | Shows empathy and compassion in response to patient stress or distress at times during the encounter through verbal and nonverbal behaviors |  | Consistently displays empathy and compassion in response to patient’s stress or distress throughout (verbal and nonverbal behaviors) |
| 1. **Honesty/Integrity** | Is not honest or offers frequent false reassurance |  | Is honest with patient and only offers false reassurance at times |  | Is consistently honest with patient and does not offer false reassurance including in challenging situations |
| 1. **Responsibility**   **/Accountability** | Blames the patient and does not foster a sense of joint responsibility for the patient’s health |  | Develops a sense of joint responsibility for the patient’s health at times |  | Develops a sense of joint responsibility for the patient’s health, including in all challenging situations |
| 1. **Promoted a Collaborative Environment** | Offers no invitation for me to speak about my prior or current ideas/solutions for change |  | Solicits my ideas about change at times and/or included me when developing a plan of action at times |  | Consistently solicits my ideas when discussing prior or current change and maintained a "partner" approach to care at all times |
| 1. **Lack of Bias** | Exhibits bias on the basis of weight, sex or ethnicity |  | Exhibits lack of bias when interacting with me |  | Exhibits lack of bias when interacting with the patient including in challenging situations |

^*^Item added after mid-OSCE feedback

**Supplemental Figure 5. History taking checklist for resident self-assessment**

Participant Study Number: ______

|  | **0** | **1** |
| --- | --- | --- |
| 1. I asked when the patient first began struggling with weight | No | Yes |
| 1. I asked the patient’s highest and lowest weights | No | Yes |
| 1. I asked about the patient’s past attempts to lose weight | No | Yes |
| 1. I asked why the patient thinks he/she is gaining/gained weight | No | Yes |
| 1. I asked a 24-hour diet recall | No | Yes |
| 1. I asked about beverage consumption | No | Yes |
| 1. I asked about prior attempts to change his/her diet | No | Yes |
| 1. I asked about barriers to healthy eating | No | Yes |
| 1. I asked about the type of physical activity the patient performs | No | Yes |
| 1. I asked about the amount of physical activity the patient performs | No | Yes |
| 1. I asked about barriers to adequate physical activity | No | Yes |
| 1. I asked about the patient’s concerns regarding his/her excess weight | No | Yes |
| 1. I asked if the patient feels supported by his/her family or partner | No | Yes |
| 1. I completed the encounter in the allotted time. | No | Yes |

**Supplemental Figure 6. Communication checklist for resident self-assessment**

Participant Study Number: _______

|  | **1** | **2** | **3** | **4** | **5** |
| --- | --- | --- | --- | --- | --- |
| 1. Language and Vocabulary | I used medical jargon frequently without explanation or used slang that was jarring. |  | I usually explained medical terms when using them; I could always explain terms if prompted. |  | I avoided medical terminology or explained without prompting, I adjusted information to suit patient’s level of understanding. |
| 1. Attention | I seemed to ignore or forget information provided by patient, often repeated questions |  | I asked/duplicated very few questions and usually explained or acknowledged repeated questions |  | I did not repeat questions without explanation or acknowledgement. Questions built off information the patient provided. |
| 1. Verbalizes understanding of history | I did not verbalize my understanding of the patient’s history. |  | I reflected, clarified, or summarized the history at times during the interview. |  | I reflected, clarified, or summarized the history frequently during the interview. |
| 1. Open Ended Questions | I exclusively used closed ended questions/did not use facilitating remarks^*^ |  | I used facilitating remarks or open-ended questions at points during the interview |  | I used many facilitating remarks and open-ended questions throughout the interview |
| 1. People First Language | I rarely used people first language |  | I used people first language at times during the interview |  | I almost always used people first language during the interview |
| 1. Organization | The encounter was disorganized. I did not use transition phrases or sign-posting^‡^ |  | The encounter was mostly organized. I occasionally used transition phrases and sign-posting |  | The encounter flowed easily and logically from one topic to the other, facilitated by the consistent use of transition phrases and sign-posting |

**Description of Open Ended Questions**

- Open ended questions are those that cannot be answered with a “yes” or “no” response and encourage elaborative responses from the interviewee
- Examples of “facilitative remarks” include: “Tell me more about that;” “What else has been happening?”

**Description of “people first language”**

- “People first language” refers to a sentence structure that avoids labeling and defining individuals by their medical condition by naming patients first and their diagnoses second. Using this sentence structure helps separate patients from their diagnoses and allows providers to focus on their patient as a whole, rather than defining them by their diagnoses.

| Examples of people first language | Examples of non-people first language |
| --- | --- |
| - “patients living with obesity,” “patients with excess weight” - “How long have you struggled with your weight?” - “Why do you think you struggle with your weight?” - “Are there any other family members with obesity?” | - “obese people,” “fat people” - “How long have you been/when did you first become obese?” - “Why do you think you are obese/fat?” - “Are there any other obese family members?” |

**Description of sign posting**

- Language used by the provider to inform the patient what will be said or done next. For example “Now that we have talked about xxx, we will discuss xxx next…”

**Supplemental Figure 7. Professionalism checklist for resident self-assessment**

Participant Study Number: _____

|  | **1** | **2** | **3** | **4** | **5** |
| --- | --- | --- | --- | --- | --- |
| 1. Respect | I lacked professional demeanor during history (e.g. minimally acknowledged patient beyond chief concern or often touched before talking) |  | I usually acknowledged the patient beyond chief concern and used talk before touch |  | I consistently interacted with patient using utmost respect (e.g. showed a lot of interest in patient beyond chief concern; consistently talked before touch) |
| 1. Empathy | I did not offer verbal or nonverbal empathetic responses to patient’s stress or distress |  | I showed empathy and compassion in response to patient stress or distress at times during the encounter through verbal and nonverbal behaviors |  | I consistently displayed empathy and compassion in response to patient’s stress or distress throughout (verbal and nonverbal behaviors) |
| 1. Honesty/Integrity | I was not honest or offered frequent false reassurance |  | I was honest with patient and only offered false reassurance at times |  | I was consistently honest with patient and did not offer false reassurance including in challenging situations |
| 1. Responsibility   /Accountability | I blamed the patient and did not foster a sense of joint responsibility for the patient’s health |  | I developed a sense of joint responsibility for the patient’s health at times |  | I developed a sense of joint responsibility for the patient’s health, including in all challenging situations |
| 1. Promoted a Collaborative Environment | I offered no invitation for the patient to speak about his/her prior or current ideas/solutions for change |  | I solicited the patient’s ideas about change at times and/or included him/her when developing a plan of action at times |  | I consistently solicited the patient’s ideas when discussing prior or current change and maintained a "partner" approach to care at all times |
